# Four-year pain relief after coblation combined with active exercise for cervical discogenic pain: Case report

**DOI:** 10.1097/MD.0000000000016409

**Published:** 2019-07-12

**Authors:** Xiuhua Li, Liqiang Yang, Jiaxiang Ni, Yuqi Zhang

**Affiliations:** a School of Medicine, Tsinghua University, Haidian District; b Department of Pain Management, Xuanwu Hospital of Capital Medical University; c Clinical Neuroscience Institute, Yuquan Hospital, Medical Center, Tsinghua University, Beijing, P.R. China.

**Keywords:** active exercise, case report, cervical lordosis, coblation, discogenic pain

## Abstract

**Rationale:**

Coblation of intervertebral disc is an effective and safe minimally invasive technology for treating discogenic pain. The inactivation of neural ingrowth around annulus and tissue ablation and coagulation are currently considered to be the major causes for success of this treatment. However, it has been found by clinical researchers that its long-term clinical outcome is not optimistic. This report has given us favorable information that this situation can be improved with multimodal therapy.

**Patient concerns:**

A 61-year-old man presented with right severe neck and shoulder pain in 2014 which could not be relieved by medications.

**Diagnoses:**

According to his symptoms and signs, this patient was diagnosed with cervical discogenic pain. And discography confirmed the diagnosis.

**Interventions:**

The patient underwent coblation of cervical intervertebral disc 4–5 (C4–5) and got apparently pain relief after surgery. After 1 month, he began to perform active exercise at least 30 min every day.

**Outcomes:**

The right neck and shoulder pain completely relieved for 4 years. The cervical lordosis of this patient was restored in 2018 which was confirmed by MRI compared in 2014 and NDI (neck disability index) decreased from 58 to 10%.

**Lessons:**

This report demonstrated that it was important and essential for clinicians to educate patients with discogenic pain to perform active exercise after minimally invasive surgery.

## 1 Introduction

Cervical and lumbar spines are easy to suffer a higher percentage of degenerated discs and subsequent discogenic pain syndrome.^[1]^ It is generally assumed that discogenic pain originates from internal disk disruption and protrusion and is mainly mediated by the sinuvertebral nerve.^[2]^ Its fundamental pathogenesis is disc degeneration and nociceptive nerve ingrowth in the outer annular of intervertebral discs.^[3,4]^ Conservative treatments such as physiotherapy, medication, and epidural injections are usually performed in patients with cervical discogenic pain, but still many patients have refractory neck and shoulder pain.^[5,6]^ Coblation technique has been applied in the pain management such as discogenic pain and neuropathic pain which has got effective therapeutic results.^[7,8]^ The long-term efficacy of coblation for discogenic pain is uncertain, but coblation nucleoplasty was a safe, effective, and slightly uncomfortable procedure.^[9,10]^

When chronic neck pain occurred, exercise or physiotherapy was usually chosen to relieve neck pain.^[11]^ Patients could benefit from the intervention but did not experience consistent reduction of neck pain. In this study, the patient underwent coblation nucleoplasty and then performed active exercise after 1 month, the symptoms were examined with a 4-year follow up.

## 2 Presenting concerns

A 61-year-old, right-handed native man visited to pain clinic with severe neck and shoulder pain for 1 year in 2014. He had sedentary lifestyle for office working and speculating in the stock market. The patient complained of right neck and shoulder pain with consistent sour swell pain, especially at night. The pain could be aggravated by holding one position with pain VAS 7–8 out of 10 and NDI (neck disability index) was 58%. Medications and local injection were invalid for pain relief. Physical examination: cervical lordosis disappeared, muscular stiffness, poor cervical motivation, right C4–5 paravertebral pressing pain positive. The power of hand gripping was normal. Cervical magnetic resonance showed C4–5 and C5–6 protrusion. Previous history: hypertension and hyperlipidemia under good control. Before surgery, the patient signed the written informed content. After confirming the target disc through discography, the patient underwent the coblation technique for C4–5 nucleoplasty by CT guided. The pain was significantly decreased with VAS 2–3 out of 10 and NDI was 16% on the third day after surgery.

In June 2018, the patient visited to pain clinic again and complained of dizzy and palpitation for 2 months. During the 4 years he had no complaints about neck and shoulder pain and NDI was 10%. The patient accepted the cervical magnetic resonance re-examination. The uncomfortable symptoms disappeared after seven blocks of cervical sympathetic nerve (once every day) (see Table 1). The ethics committee of Yuquan Hospital, Tsinghua University approved the study (ethical approval number: 20190003).

**Table 1:**
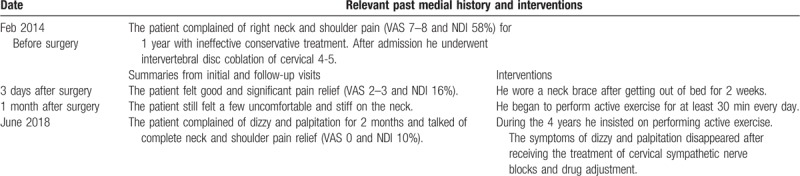
Timeline of this case report.

## 3 Clinical findings

Before surgery VAS of pain was 7–8 and NDI was 58%; third day after surgery VAS 2–3 and NDI 16%; follow-up for 4 years VAS 0–1 and NDI 10%.

One month after surgery, the patient began to perform active exercise included squatting up and down and jogging at least 30 min every day. He adapted an active lifestyle.

**Figure 1:**
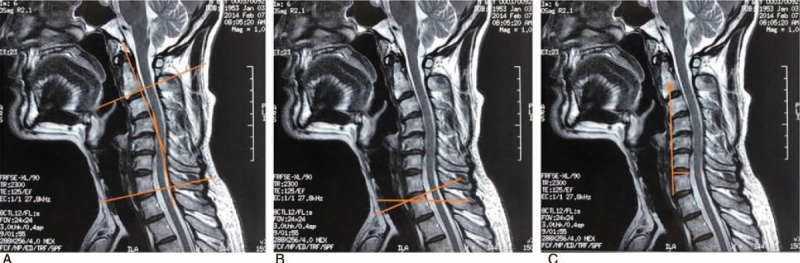
Preoperative neutral sagittal view of T2-weighted image in 2014. (A) showed C2–C7 Cobb angle; (B) showed the angle of T1 slope; (C) showed C2–C7 sagittal vertical axis.

**Figure 2:**
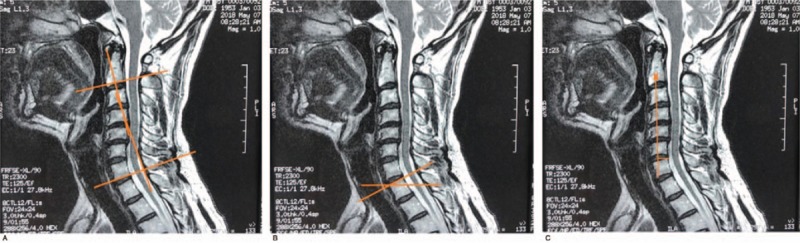
Preoperative neutral sagittal view of T2-weighted image in 2018. (A) showed C2–C7 Cobb angle; (B) showed the angle of T1 slope; (C) showed C2–C7 sagittal vertical axis.

Comparison of cervical lordosis between pre- and post-operation: It showed that SVA was 1.3 cm, C2–C7 Cobb angle was −10°, and the angle of T1S was 23° by neutral sagittal view of T2-weighted image in 2014 before surgery (see Fig. 1). By neutral sagittal view of T2-weighted image in 2018 after surgery, it showed that SVA was 0.9 cm, C2–C7 Cobb angle was 10°, and the angle of T1S was 28° (see Fig. 2). Two doctors measured the numerical values and then took the average.

## 4 Diagnostic focus and assessment

1. Diagnostic methods: discography and cervical MRI;
2. There were no diagnostic challenge and the patient was cooperative and got positive therapy;
3. Diagnostic reasoning: it was definite to diagnose according to pain distribution, pain symptoms and image data and no other diagnosis was considered.
4. Prognostic characteristics: the neck and shoulder pain disappeared and NDI improved during the 4 years.

### 4.1 Therapeutic Focus and Assessment

The types of interventions were minimally invasive surgery and active exercise for 30 min after surgery every day. Pain VAS, NDI, and cervical MRI were observed after surgery.

### 4.2 Follow-up and Outcomes

During the 4 years after C4–5 coblation the patient got complete pain relief, decreased NDI and the restoration of cervical lordosis in 2018 compared with that in 2014. The patient was not under any alternative therapy for his neck pain except for performing active exercise. No side effects occurred during the 4 years.

## 5 Discussion

Cervical discogenic pain has long been recognized as a common source of neck pain and disability which resulting from herniation or degeneration of the cervical intervertebral disc.^[12]^ The spinal discogenic problem could change the distribution of axial load from the posterior column to anterior as lordosis lost and further accelerated the disc degeneration which produce pain and disability.^[1,13]^ The change of cervical lordotic curve was usually found by cervical spine images in clinic.^[3,14]^ MRI has the superiority because of no radiation injury for human bodies compared with X-ray and CT.^[15–17]^ In this report we choose the frequently-used and good repeated methods to evaluate the cervical lordotic curvature including SVA, C2–C7 Cobb and T1S.^[18,19]^ It has reported that C2–C7 SVA showed a positive correlation with NDI which was also certified in this study.^[20–22]^

In this report, the cervical lordotic curve was abnormal and reverse in 2014 before surgery. Measuring the SVA (1.3 cm) and reverse C2–C7 Cobb angle (−10°), it could be found that cervical lordotic curvature was straightened and emerged the S shape change. The angle of T1S was 23° (<26°) which further showed the loss of cervical lordotic curvature. When cervical lordotic curvature changed, it was a long process to restore for conservative treatment, and nobody knew the exact time. It was pleasantly found in this study that the abnormal cervical lordosis improved in 2018. For SVA (0.9 cm) in 2018, it was shorter compared with that in 2014. The angle of T1S was 28° (>26°) and positive C2–C7 Cobb angle (10°) further showed the cervical lordotic curvature near the normal. This study also demonstrated that T1S was positively correlated with C2–C7 Cobb.

The patient was not under any alternative therapy during the 4 years except to take medicine for stable blood pressure because of his hypertension. He adapted an active lifestyle and insisted on squatting up and down and jogging at least 30 min every day after surgery. He changed his poor habit style (once playing for 2 h with no movement) and NDI decreased apparently. An exercise program focusing on cervical extensor muscle strengthening and restoring the balance of flexor and extensor muscles was recommended for patients with loss of cervical lordosis.^[12,23]^ Some scholars also verified the importance of exercise who reported that exercise was related with durable slight to moderate improvements in function and chronic pain.^[24]^ Chung also reported that neck isometric exercise and craniocervical flexion exercise could improve pain, NDI and active cervical range of motion, moreover, craniocervical flexion exercise could improve absolute rotation angle of cervical lordosis.^[25]^ The result suggested that squatting up and down and jogging might help to improve chronic neck pain and restore cervical curvature.

According to the published literatures a single technique did not yield good clinical results no matter coblation or active exercise for chronic neck pain.^[26,27]^ In this study the patient performed active exercise every day after minimally invasive surgery and got long-term pain relief which gave us clear strategy of clinical treatment for patients with discogenic pain. Passive exercise was usually accepted easily by people when they felt uncomfortable in clinic. Sometimes when they received an operation, they were often afraid of active exercise such as spinal surgery. The results from this report showed that active exercise and multimodal therapy produced more beneficial outcomes. It was consistent with the previous reports.^[28,29]^ According to this report, patients should know the importance of performing active exercise for postoperative rehabilitation care.

## 6 Conclusion

In this study clinical decision-making was provided for patients with cervical discogenic pain even accompanying abnormal cervical lordosis. Coblation nucleoplasty could solve the discogenic pain in a short time and postoperative active exercise prolonged the curative effect. It was very important to educate patients about the importance of active exercise after surgery. However, this study has its limitation because it is a single case study. Further study involving more cases is needed to clearly elucidate the effects of active exercise for cervical discogenic pain with the treatment of coblation.

## 7 Patient perspective

The patient shared his experience of his care in three periods: On the third day after surgery he felt comfortable and apparent pain relief of neck and shoulder; a month later he took off his neck holder and began to perform active exercise. During the 4 years he felt complete pain relief and was satisfied with the treatments.

## Acknowledgment

No financial benefits were provided to the authors. No conflicts of interest were in this study.

## Author contributions

**Methodology:** Jiaxiang Ni.

**Project administration:** Liqiang Yang.

**Writing – original draft:** Xiuhua li.

**Writing – review & editing:** Yuqi Zhang.
